# Pharmacokinetic studies of [^68^ Ga]Ga-PSMA-11 in patients with biochemical recurrence of prostate cancer: detection, differences in temporal distribution and kinetic modelling by tissue type

**DOI:** 10.1007/s00259-021-05420-1

**Published:** 2021-06-10

**Authors:** Dimitrios S. Strauss, C. Sachpekidis, K. Kopka, L. Pan, U. Haberkorn, A. Dimitrakopoulou-Strauss

**Affiliations:** 1 Clinical Cooperation Unit Nuclear Medicine, German Cancer Research Center (DKFZ), Im Neuenheimer Feld 280, 69210 Heidelberg, Germany; 2 Institute of Radiopharmaceutical Cancer Research, Helmholtz Zentrum Dresden Rossendorf, Dresden, Germany; 3 German Cancer Consortium (DKTK), Heidelberg and partner site Dresden, Germany; 4 Fakultät Chemie und Lebensmittelchemie, Technische Universität Dresden, Dresden, Germany; 5 Division of Nuclear Medicine, University of Heidelberg, Heidelberg, Germany

**Keywords:** Ga-PSMA, PSMA, PSMA Kinetic, Recurrent prostate cancer

## Abstract

**Purpose:**

[^68^ Ga]Ga-PSMA-11 is a promising radiopharmaceutical for detecting tumour lesions in prostate cancer, but knowledge of the pharmacokinetics is limited. Dynamic PET-CT was performed to investigate the tumour detection and differences in temporal distribution, as well as in kinetic modelling of [^68^ Ga]Ga-PSMA-11 by tissue type.

**Methods:**

Dynamic PET-CT over the lower abdomen and static whole-body PET-CT 80–90 min p.i. from 142 patients with biochemical recurrence were retrospectively analysed. Detection rates were compared to PSA levels. Average time-activity curves were calculated from tumour lesions and normal tissue. A three-compartment model and non-compartment model were used to calculate tumour kinetics.

**Results:**

Overall detection rate was 70.42%, and in patients with PSA > 0.4 ng/mL 76.67%. All tumour lesions presented the steepest standardised uptake value (SUV) incline in the first 7–8 min before decreasing to different degrees. Normal tissue presented with a low uptake, except for the bladder, which accumulated activity the steepest 15–16 min. p.i.. While all tumour lesions continuously increased, bone metastases showed the steepest decline, resulting in a significantly lower SUV than lymph node metastases (60 and 80–90 min). Transport rate from the blood and tracer binding and internalisation rate were lower in bone metastases. Heterogeneity (fractal dimension) and vascular density were significantly lower in bone metastases.

**Conclusion:**

Even at low PSA between 0.51 and 0.99 ng/mL, detection rate was 57%. Dynamic imaging showed a time window in the first 10 min where tumour uptake is high, but no bladder activity is measured, aiding accuracy in distinction of local recurrence. Kinetic modelling provided additional information for tumour characterisation by tissue type.

**Supplementary Information:**

The online version contains supplementary material available at 10.1007/s00259-021-05420-1.

## Introduction

In the diagnosis of prostate cancer (PC) recurrence, the detection of tumour lesions is essentially based on risk assessments using tumour markers and clinical information, as conventional imaging (computed tomography (CT), magnetic resonance imaging (MRI), transrectal ultrasound, bone scintigraphy) shows insufficient performance in depicting a progression of the disease. An increase in the prostate-specific antigen (PSA) after initial treatment is referred to as a biochemical recurrence (BCR) and indicates a progression of the disease. However, this PSA increase precedes a clinically manifest metastasis by an average of 7 to 8 years, whereby only 11 to 14% of patients with biochemical recurrence even show a detectable tumour lesion in CT. PSA may also be elevated in the natural course after therapy, making the presence of BCR a highly variable risk factor for prostate cancer mortality, which complicates diagnosis and therapy. Patients with biochemical recurrence who receive curative, local therapy particularly early benefit best from this. However, it is in this group of patients with PSA < 1 that conventional imaging techniques fail most in detecting tumour lesions. At the same time, patients with metastatic disease benefit less from local therapy, as they require systemic therapy. The detection of tumour lesions in biochemical recurrence is therefore essential to distinguish between local and systemic progression [1]. Since its first application in 2011, imaging with [^68^ Ga]Ga-PSMA-11 PET-CT in prostate cancer has proven to be a potential game changer in recurrence diagnostics [2–8].

Knowledge about the pharmacokinetics of [^68^ Ga]Ga-PSMA-11 in PET-CT is still limited, as static acquisition limits distributional information to a single time point. This study has three major goals: First, to demonstrate the detection of tumour lesions with [^68^ Ga]Ga-PSMA-11 PET-CT in biochemical recurrence of prostate carcinoma. Secondly, to illustrate the temporal distribution of [^68^ Ga]Ga-PSMA-11 in tumour lesions and normal tissue. Third, differences between tumour lesions will be assessed using a kinetic model (compartment and non-compartment). The primary objective is to find differences between lymph node metastases and bone metastases in terms of standardised uptake value (SUV), heterogeneity (FD), vascular density (V_B_), receptor binding and internalisation rate (k_3_), and transport rate from blood (K_1_).

## Materials and methods

A total of 160 [^68^ Ga]Ga-PSMA-11 PET-CT scans from 142 patients were retrospectively evaluated, excluding patients without any initial therapy. Patients after radical prostatectomy (RP) were counted as BCR as soon as the PSA was detectable (> 0.004 ng/mL). Eighteen PET-CT follow-up examinations were excluded from the quantitative and statistical analysis in order to avoid bias. A small number of patients have already been published in other studies [9, 10]. This analysis was performed in line with the principles of the Declaration of Helsinki and has been approved by the Ethical Committee of the University of Heidelberg (S-253/2019). All subjects signed an informed consent form.

Gallium-68 was eluted on site from a germanium-68 generator and subsequently bound to the urea-based prostate-specific membrane antigen (PSMA) inhibitor PSMA-HBED-CC, namely PSMA-11, bearing the targeting vector Glu-urea-Lys using a radiosynthesizer as already described [11, 12].

**Fig. 1:**
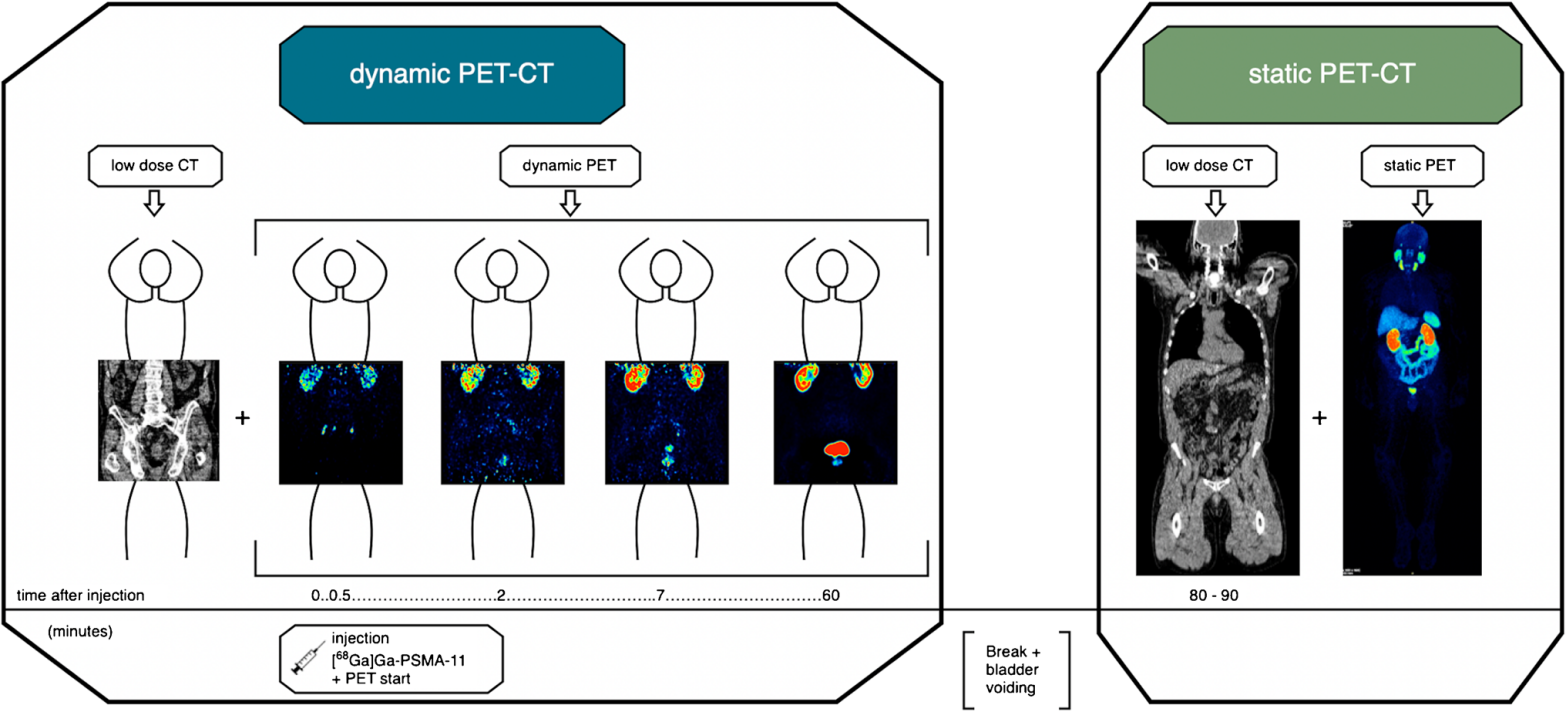
Acquisition protocol of the study. Left box: dynamic PET-CT of the pelvis and lower abdomen. After an initial low-dose CT (left image), the dynamic PET starts with injection of [^68^ Ga]Ga-PSMA-11 (timeline 0 min). The four PET images are arranged chronologically (left to right): after 0.5 min, 2 min, 7 min and 60 min p.i. After 60 min, the acquisition is finished and the patient goes to the toilet to empty the bladder, which is shown here as the period between the two boxes in the middle. Right box: static whole-body PET-CT 80–90 min p.i.. A low-dose CT is performed over the entire body, a part of which is shown here as an example in the left image of the right box. This is followed directly by the whole-body PET-CT image, which is shown as MIP in the right image of the right box. All images shown are in coronal slicing

The acquisition protocol (Fig. 1) was recorded with a Siemens Biograph mCT (TrueV configuration FOV 22.1 cm, HD-PET) using low-dose CT (128 detector rows, 120 keV, 30 mAs). The PET protocol was specially customised in order to dynamically acquire two bed positions (pelvis and lower abdomen) alternately, thus doubling the FOV (44.2 cm). The dynamic acquisition consisted of 24 frames (ten frames each 30 s, five frames each 60 s, five frames each 120 s and four frames each 600 s). Static PET had an image duration of 2 min per bed position. 400 × 400 pixels PET images were iteratively reconstructed using an ordered subset expectation maximisation (OSEM) algorithm, which divided the data into 12 subsets with 6 iterations to accelerate calculation [13].

Qualitatively, contrast and distinctiveness were assessed with the Aycan workstation 3.14.006. Quantitatively, five tumour lesions (categorised as local recurrence, lymph node metastases, bone metastases and other metastases) per patient were selected that were preferably visible in dynamic and static PET for comparability and could be well-differentiated using PMOD 3.7. A lesion in PET was considered pathological if it showed a morphological correlate in CT, which did not correspond to a structure with physiological PSMA uptake. Therefore, PSMA avid but normal-sized lymph nodes were considered pathological. Known pitfalls, like sympathetic ganglia and bone fractures, were taken into consideration [14]. Four normal tissues were selected in the dynamic images (input, bladder, colon, m. gluteus). Lesions were assessed with a volume of interest (VOI) in isocontour mode (pseudosnake). The most important quantitative value in PET is SUV, setting the measured activity of a lesion in relation to the applied activity and body weight [15]. SUVs early were measured in the last 10 min of the dynamic acquisition, SUV late in the static PET 80–90 min p.i.. The results were evaluated by two experienced nuclear medicine physicians (CS, ADS).

**Fig. 2:**
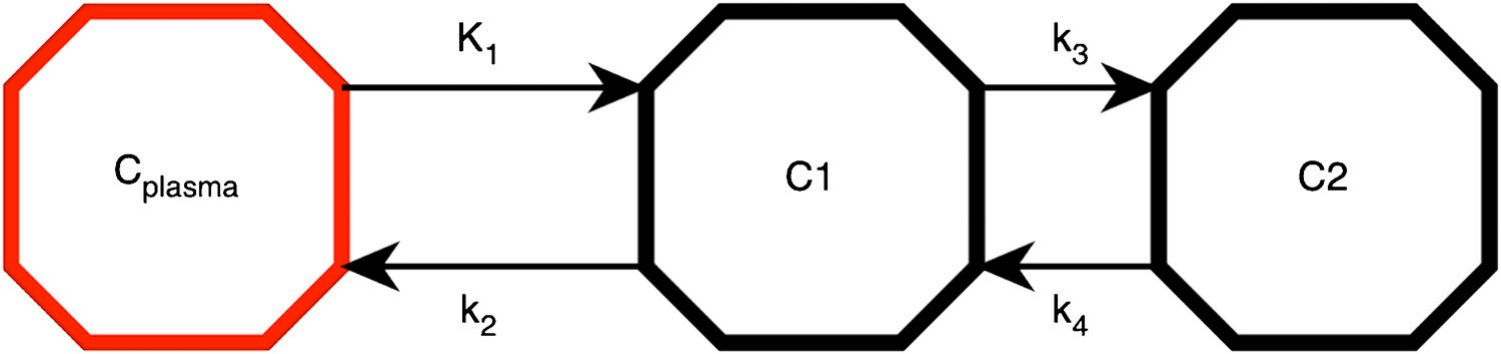
The three-compartment model: the red compartment C_plasma_ represents the tracer activity in the blood. Compartment C1 (middle octagon) represents unspecific bound and free tracer parts in the tissue. K_1_ is the transport rate of the tracer from the blood into the tissue. k_2_ correspondingly stands for the backward path, or efflux. The C2 compartment (right octagon) stands for PSMA-bound and clathrin-mediated internalised tracer components. K_3_ measures the receptor binding and internalisation rate and k_4_ in turn measures the externalisation rate. The unit of measurement for all K values is 1/min in each case

From the dynamic PET, mean time-activity curves and in addition kinetic parameters were calculated by means of a three-compartment model (Fig. 2). Kinetic models derived from pharmacology are an adaptable tool for dividing physiological steps into subsystems in order to represent biological processes in a simplified way [16–18]. The individual compartments are tracer parts that behave uniformly and interact with each other. The gold standard to measure the tracer flow is a periodic arterial blood sample parallel to the PET. A simpler and more efficient way is to retrieve the input function from the VOI of an arterial vessel [19, 20]. The quality of the input function is user-dependent, as it is crucial to set a narrow activity peak by means of a narrow VOI. The common iliac artery was chosen for the input function; depending on the representability, the abdominal aorta was used. In order to avoid overfitting in iterative fitting of the kinetics, which would increase image noise, and to make the methodology less user-dependent, the PKIN module in PMOD was modified by our research group at DKFZ: A machine learning algorithm approximated the iterative fitting using a database of confirmed tumour lesion data to find a first approximation of the kinetic parameters based on the quality of the given database. In the second step, the curve fit was performed using the classical Levenberg-Marquardt algorithm, depending on the quality of the fit (based on the Akaike criterion and the chi-square value), as presented in previous studies [20, 21]. Besides K_1_ to k_4_, the vessel density V_B_ (fractional blood volume) was also calculated. As a quality criterion, the model was accepted if 0 < V_B_ < 1 and K_1_, k_2_, k_3_ and k_4_ were < 1.

In addition to the compartment modelling, a non-compartment analysis was carried out, by means of which the fractal dimension (FD) was calculated from the time-activity data for each pathological VOI according to the so-called box-counting principle. The FD is a dimensionless parameter, with values ranging between 0 and 2 in a two-dimensional space, used to describe the temporal heterogeneity of activity accumulation within a VOI. Values close to 0 thus show a deterministic distribution, values close to 2 correspondingly a chaotic distribution of the tracer activity over the temporal measurement [20, 22].

The statistical analysis was done with Stata/MP 14.2 (StataCorp LLC). The detection rate was evaluated in comparison to PSA levels. To validate the kinetic parameters, a Spearman rank correlation analysis was performed with the SUV early. After testing for normal distribution (histogram, Shapiro–Wilk W test), a Wilcoxon rank sum test (5% significance level) was chosen to verify the research hypothesis. In order not to generate α-error inflation, as well as to maintain the significance level at good power, the testing of differences between lymph node metastases and bone metastases was done a priori: SUVmean early, SUVmax early, SUVmean late, SUVmax late, FD, V_B_, k_3_ and K_1_.

## Results

[^68^ Ga]Ga-PSMA-11 PET-CT provided a subjectively good image quality with high contrast of the tumour lesions at very low background activity. The detection rate in the entire collective was 70.42% (100/142) with a median PSA of 2.3 ng/mL (reported in 137/142 patients). A total of 40 patients (28.2%) had a PSA < 1, and 11 patients (7.8%) after radical prostatectomy had a PSA < 0.2. The median Gleason score was 7 (reported in 97/142 patients); the applied activity averaged 193.6 MBq ± 62.87. In the subgroup of patients with PSA < 1, the detection rate was 45% (18/40), in patients with 0.2 < PSA < 1 and 0.4 < PSA < 1, the detection rate was 50% (14/28) and 55.56% (10/18), respectively. Patients with a PSA increase to > 2 ng/mL showed a higher detection rate of 83.75% (67/80).

Patients with a pathological PET-CT had a higher PSA than patients with a negative PET-CT (median 3.5 vs. 0.72). The Gleason score was the same in both groups (median 7).

The static whole-body PET-CT images showed a physiologically filled bladder with visualisation of the kidneys and ureters, as well as accumulation of the salivary glands, liver, spleen and intestinal parts in all patients. A total of 272 tumour lesions were selected, including 23 local recurrences, 181 lymph node metastases, 61 bone metastases and seven other metastases (soft tissue and lung). All tumour lesions of the dynamic images were also visible in the static images.

**Fig. 3:**
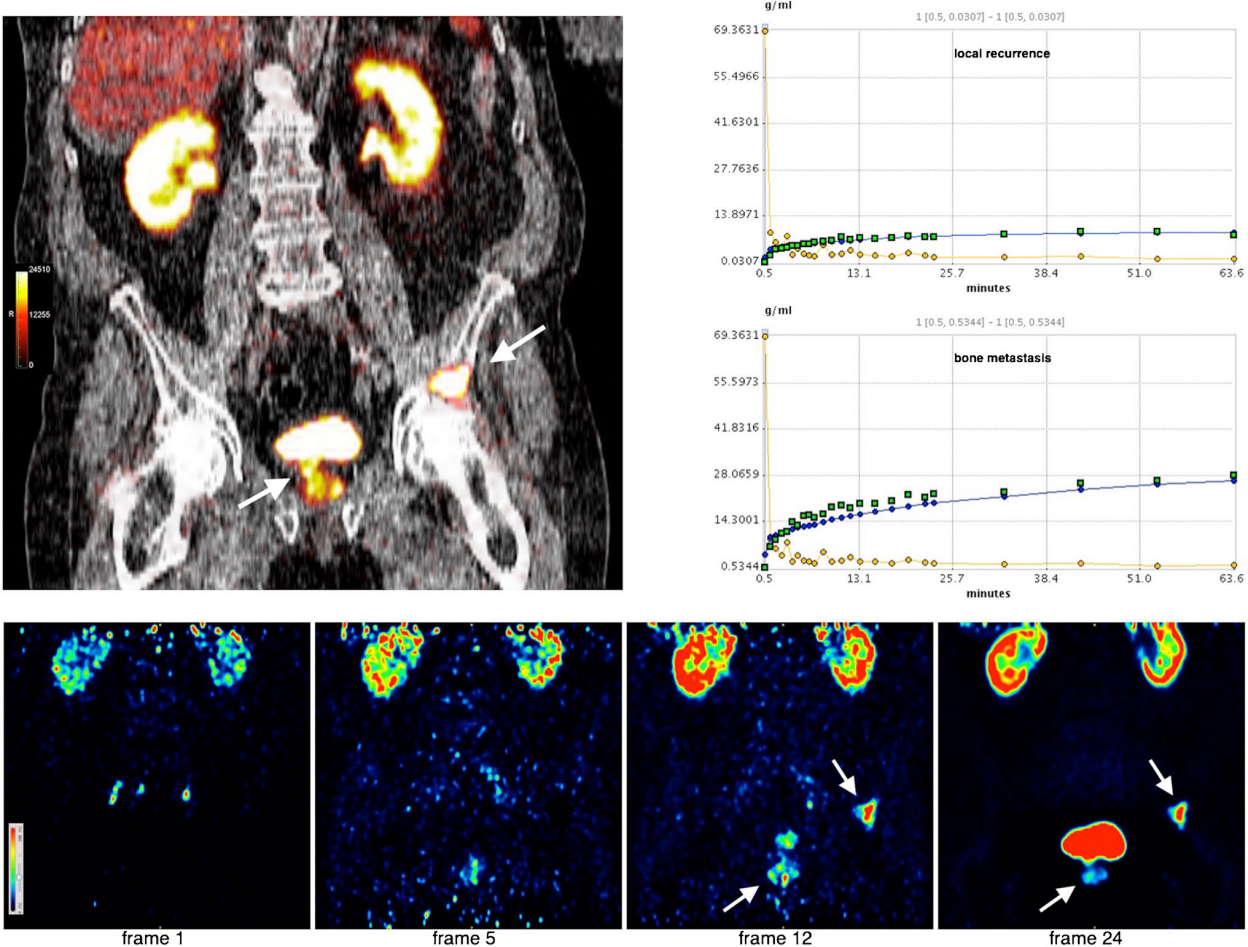
Dynamic PET-CT of a BCR (PSA 2.47 ng/mL) after radiation and hormone therapy with local recurrence (arrow) and bone metastasis (arrow). Top left: Fused coronary PET-CT image showing the local recurrence and bone metastasis in the os ilium 60 min p.i.. Top right: Mean time-activity curves in the local recurrence (top diagram) and the bone metastasis (bottom diagram) compared to the vascular activity in each case. Bottom row: Coronary PET images in time sequence from frame 1 (30 s after injection) to frame 24 (60 min after injection). Frame 5 (= 2.5 min p.i.) already shows accumulation in the local recurrence and bone metastasis, without bladder activity being visible. Frame 12 (= 7 min p.i.) shows increasing activity in all lesions and at the same time activity now appears in the bladder. Frame 24 (= after 60 min) shows strong activity in the bladder adjacent to the local recurrence which is barely increasing in activity

**Fig. 4:**
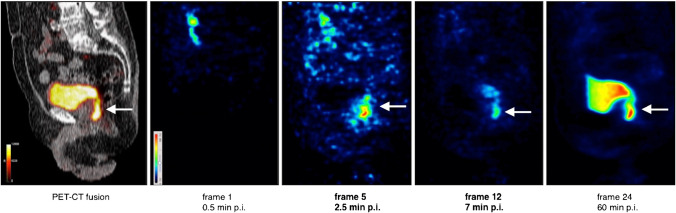
Illustration of a local recurrence (arrow) near the urinary bladder. Far left: Fused sagittal PET-CT image 60 min p.i. showing the local recurrence dorsal to the bladder. Right: Sagittal PET images in temporal sequence from frame 1 (30 s after injection) to frame 24 (60 min after injection)

**Fig. 5:**
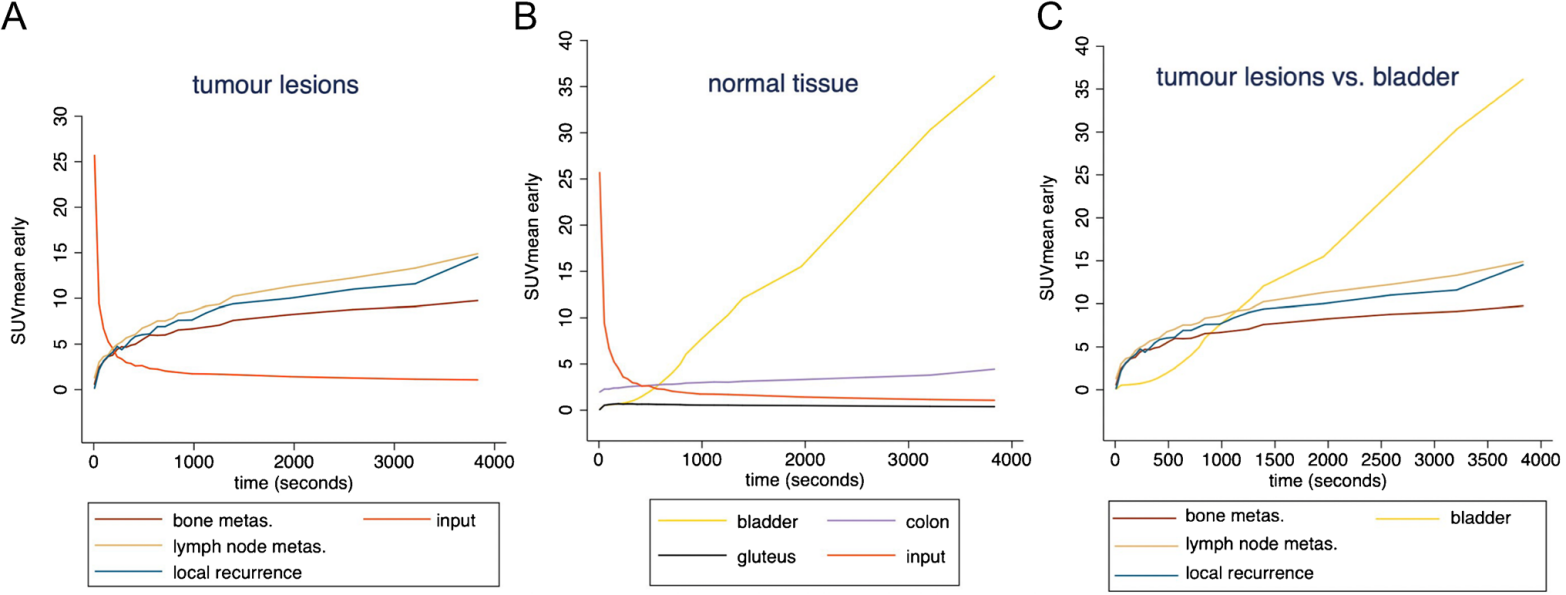
Mean time-activity curves of tumour lesions (left diagram **A**) and normal tissue (middle diagram **B**) vs. input (red curve). TAC of tumour lesions vs. bladder activity (gold curve) is shown in the right diagram (**C**). Tumour lesions showed the steepest incline within the first few minutes, before flattening out to different degrees while maintaining a continuous raising uptake. Normal tissue showed a low and plateau-like uptake, except for the bladder which began accumulating slowly after 7 to 8 min p.i. and showed the steepest incline of all lesions 15 min p.i

From the dynamic PET-CT images, 226 tumour lesions in the pelvis and lower abdomen could be quantitatively evaluated (Table 1), including 23 local recurrences, 168 lymph node metastases, 34 bone metastases and one other metastasis (soft tissue). In total, 452 VOIs were drawn in normal tissue, of which 113 were input, 113 colon, 113 bladder and 113 gluteal muscle (Table 2). Local recurrences near the bladder were easier to differentiate in the early dynamic frames, as shown in Figs. 3 and 4. The mean time-activity curves (TACs) in Fig. 5 showed that tumour lesions had an extremely steep increase in activity within the first few minutes, which flattened out to varying degrees but remained continuously increasing. The arterial vessels had the highest activity from the beginning of the measurement, with the input falling extremely steeply within the first minutes. The bladder only showed a flat ascent after 7 to 8 min, which increased slowly. Then, 15 to 16 min p.i., the bladder showed the strongest increase of all detected lesions, which at this point had reached the SUV level of tumour lesions and was continuously increasing. The colon had a low and hardly changed uptake over time. The background activity in the gluteus muscle was extremely low and plateau-like.

**Table 1:** SUV and kinetic parameters for tumour lesions in dynamic PET, including 23 local recurrences, 168 lymph node metastases, 34 bone metastases and one other metastasis

	*Local recurrence*		*Lymph node metas*		*Bone metas*		*Other metastasis*	
	Mean ± SD	Median	Mean ± SD	Median	Mean ± SD	Median	Mean ± SD	Median
*SUVmean early*	14.6 ± 14.29	8.39	15.24 ± 20.36	9.24	11.36 ± 14.6	5.63	12.31	12.31
*SUVmax early*	25.49 ± 21.41	16.54	26.39 ± 34.17	16.06	19.15 ± 23.42	8.82	23.78	23.78
*K*_*1*_	0.16 ± 0.13	0.13	0.19 ± 0.15	0.15	0.19 ± 0.2	0.11	0.18	0.18
*k*_*2*_	0.15 ± 0.2	0.08	0.2 ± 0.28	0.07	0.23 ± 0.29	0.07	0.02	0.02
*k*_*3*_	0.36 ± 0.35	0.21	0.32 ± 0.32	0.21	0.29 ± 0.31	0.19	0.02	0.03
*k*_*4*_	0.18 ± 0.31	0.02	0.14 ± 0.28	0.02	0.12 ± 0.25	0.02	5e-74	5e-74
*V*_*B*_	0.01 ± 0.02	3.3e-14	0.05 ± 0.12	5.44e-3	0.03 ± 0.07	1.3e-4	6e-04	6e-04
*FD*	1.25 ± 0.1	1.26	1.25 ± 0.12	1.28	1.22 ± 0.09	1.23	1.3	1.3

**Table 2:** SUV of normal tissue from dynamic imaging (113 VOIs in each normal tissue)

	*Bladder*		*Input*		*Colon*		*Gluteus*	
	Mean ± SD	Median	Mean ± SD	Median	Mean ± SD	Median	Mean ± SD	Median
*SUVmean early*	35.78 ± 30.62	28.42	1.03 ± 0.55	0.97	4.41 ± 2.57	3.81	0.38 ± 0.12	0.37
*SUVmax early*	50.42 ± 43.53	36.68	2.21 ± 5.05	1.38	8.24 ± 4.57	7.74	0.82 ± 0.31	0.76

**Fig. 6:**
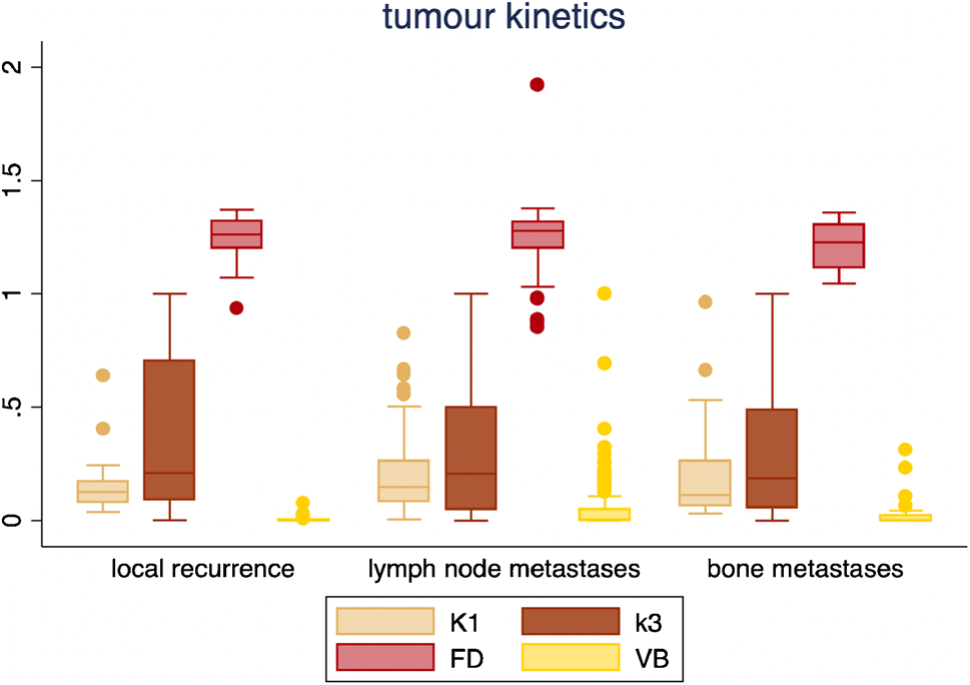
Boxplots of kinetic parameters for tumour lesions in dynamic imaging. FD is nondimensional; the other parameters are measured in 1/min

Evaluation of kinetics using the three-compartment model is shown in Fig. 6: bone metastases had a minimally lower transport rate from the blood (K_1_) compared to lymph node metastases (median 0.1 vs. 0.15). Bone metastases also showed a slightly lower receptor binding and internalisation rate than lymph node metastases (median 0.19 vs. 0.21). Vessel density, V_B_, was by far the lowest in local recurrences (median 3.3e-14) and higher in bone metastases and lymph node metastases, respectively (median 1.3e-4 vs. 5.44e-3). The fractal dimension was minimally lower in bone metastases than in lymph node metastases (median 1.23 vs. 1.28). Local recurrences, with the exception of V_B_, mostly showed kinetic parameters between the level of lymph node metastases and bone metastases with a slight tendency towards the level of lymph node metastases (Table 1).

Spearman rank correlation analysis revealed a strong correlation of the FD with the SUVmean early and SUVmax early of all tumour lesions with Rho (ρ) = 0.95 and 0.91, which was also strongly maintained in the subgroup analysis of the SUVs of the individual tumour classes (local recurrences, lymph node metastases and bone metastases) with Rho (ρ) > 0.7. V_B_ showed only a weak correlation with both SUVs of tumour lesions measured in dynamics with Rho (ρ) < 0.1. The transport rate from blood K_1_ showed a medium strong correlation with SUVmean early and SUVmax early of all tumour lesions Rho (ρ) = 0.37 and 0.38. The receptor binding and internalisation rate k_3_ also showed a medium strong correlation with SUVmean early and SUVmax early with Rho (ρ) = 0.32 and 0.31.

A Wilcoxon rank sum test was performed á priori to further refine the differences between lymph node metastases and bone metastases in all SUVs, FD, V_B_, k_3_ and K_1_. The greater decrease in the TAC of bone metastases was expressed in a significantly lower SUVmean early and SUVmax early compared to lymph node metastases (median 5.63 vs. 9.24; median 8.82 vs. 16.06; p ≤ 0.05). In the static whole-body images 80–90 min p.i., the SUVmean late and SUVmax late correspondingly were also significantly lower in bone metastases than those in lymph node metastases (median 6.03 vs. 10.63; median 11.46 vs. 19.86; p ≤ 0.05; Table 3). Likewise, FD (heterogeneity) and V_B_ (vessel density) were significantly lower in bone metastases (median 1.23 vs. 1.28; median 1.3 × 10^−4^ vs. 5.44 × 10^−3^ p ≤ 0.05). The differences in receptor binding and internalisation rate k_3_ were not significant, so to maintain the significance level, no further testing of K_1_ was carried out.

**Table 3:** SUV of tumour lesions from static imaging. The evaluation included 23 local recurrences, 181 lymph node metastases, 61 bone metastases and 7 other metastases

	*Local recurrence*		*Lymph node metas*		*Bone metas*		*Other metastases*	
	Mean ± SD	Median	Mean ± SD	Median	Mean ± SD	Median	Mean ± SD	Median
*SUVmean late*	13.11 ± 11.53	11.11	15.75 ± 18.79	10.63	10.13 ± 11.09	6.03	5.55 ± 3.96	3.91
*SUVmax late*	34.41 ± 26.97	23.19	30.22 ± 37.48	19.86	20.03 ± 22.45	11.46	12.53 ± 12.89	7.14

## Discussion

In total, 27 to 53% of all patients with prostate carcinoma develop BCR after initial therapy (radiation or radical prostatectomy), which can be an indication of progression, but also occurs in the natural course and with benign processes. Conventional imaging (CT, MRI, transrectal ultrasound, bone scintigraphy) shows poor detection rates in recurrence diagnosis, which is why clinical and histological parameters are used to assess the risk of tumour progression. In the worst case, patients with BCR undergo several imaging modalities without detectable lesions. The type and choice of therapy is very difficult because of the trade-off between toxicity if over-treated and tumour progression if under-treated. The identification of tumour lesions to detect and differentiate between local and systemic progression is therefore essential [1].

The overall detection rate (70%) was lower in this study than in the main literature, due to different patient populations. PSMA upregulation is known to depend on PSA, histological parameters and also hormone therapy [23–27]. In this collective, the median PSA was relatively low (2.3 ng/mL), and, most importantly, patients with PSA < 0.2 ng/mL were also counted as BCR, which makes detection much more difficult but also represents a realistic patient collective. Better comparable are, therefore, the detection rates of the difficult subgroup with 0.4 < PSA < 1 (56%) and PSA > 2 (84%). A study with a homogeneous patient population found an overall higher detection rate of 89.5%, which was stratified by PSA 0.5– < 1 still 72.73% [28]. Afshar-Oromieh et al. reported a detection rate of 79.5% with comparable PSA (median 2.2) in the patient population. The subgroup with PSA 0.51– ≤ 1 had a detection rate of 73% [3]. A recent meta-analysis of histopathologically validated lesions reported a detection rate for PSA < 2 and > 2 of 63% and 94%, respectively [4].

**Fig. 7:**
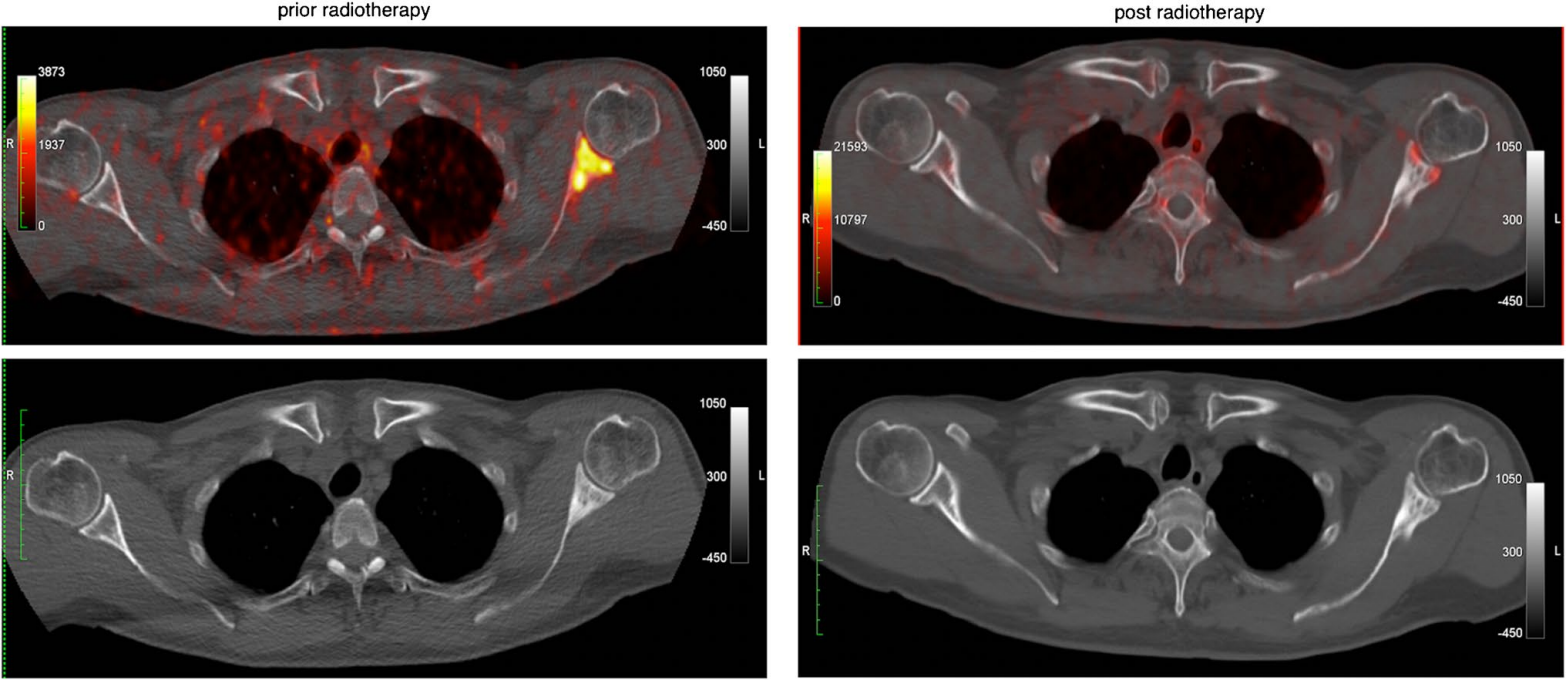
Follow-up of a patient after radiation of a shoulder metastasis in transversal section planes. The upper row shows fused PET-CT images, the lower row the native CT images. The left column shows the initial presentation with a PSA of 0.57 ng/mL after prostatectomy. This revealed a clear bone metastasis in the left scapula (SUVmax late 8.98, SUVmean late 3.09). In the native CT image at the bottom left, the bone metastasis is sclerotic. The right column shows the [^68^ Ga]Ga-PSMA-11 PET-CT images 2 years later. The shoulder metastasis has since been irradiated and no longer shows increased activity in the upper fused PET-CT image. Only a residual spot-like uptake can be seen. Osteoblastic changes are still visible on the native CT image in the lower right image. The patient came for follow-up with a rising PSA (2.49 ng/mL), and during the same examination, a local recurrence was detected in the [^68^ Ga]Ga-PSMA-11 PET-CT, which is not shown here

The detection rate of [^68^ Ga]Ga-PSMA-11 PET-CT in BCR is thus higher than in all other imaging modalities [2, 7]. CT and MRI detect lymph node metastases by size and morphology, but this is quite inaccurate because benign lymph nodes also vary greatly in size, resulting in only 2.5% of patients with lymph node metastases being detected by CT [1]. PSMA also showed the best detection rate for bone metastases in a recent meta-analysis [6]. Especially, patients with PSA rises < 0.5 ng/mL benefit from early curative salvage radiotherapy after radical prostatectomy, with 80% remaining progression-free for 5 years [1]. The strength of PSMA imaging in the future may lie in identifying these patients as well as those who require systemic therapy due to metastases. Phase-III studies are also investigating whether dose application can be optimised by adjusting the radiation field to increase efficacy and minimise side effects [29]. Molecular diagnostics using [^68^ Ga]Ga-PSMA-11 PET-CT further offers the chance of much finer therapy monitoring, as metabolic changes usually precede anatomical adaptation by a long way [30]. A persistent sclerosis of a bone metastasis after radiation on CT could be classified as a benign healing process on PET and at the same time the rising PSA could be explained by a local recurrence (Fig. 7). In another patient follow-up, [^68^ Ga]Ga-PSMA-11 PET-CT showed uptake in two known rib metastases, despite a non-detectable PSA (< 0.004 ng/mL), thus providing valuable information to the therapist.

Renal excretion is a shortcoming in [^68^ Ga]Ga-PSMA-11 imaging, which can make it difficult to delineate local recurrence. To further increase the contrast of the tumour lesions and at the same time reduce bladder activity, late imaging 3 h p.i. and furosemide administration with abundant hydration have been suggested [3]. With these measures, bladder activity could be reduced but still remained prominent with a SUVmax of 22.1 [31]. However, due to the short half-life (67.9 min), the count rate of ^68^ Ga is lower, and in one study, 21% of bone metastases even showed a reduced uptake in the late images 2.5 h p.i. [32]. A more innovative approach may be, therefore, to acquire early pelvic images in the first 5 to 10 min p.i., as tumour lesions show an extremely rapid onset even before activity is measured in the bladder. Using time window (Fig. 5C) has been postulated by several authors and the data of this analysis underline this idea [33]. Uprimny et al. were able to double the detection rate of local recurrences in 203 patients (12.8% vs. 24.6%) by static early imaging (5 min p.i.) [34]. Sachpekidis et al. were able to detect local recurrence in one out of four patients only on dynamic images in a small cohort [9]. In this study, all local recurrences were visible on static and dynamic PET, but the dynamic images aided differentiation by increasing diagnostic certainty. Acquisition protocols for [^68^ Ga]Ga-PSMA-11 PET-CT could, therefore, ideally include an additional early acquisition of the pelvis as it is convenient to integrate into the clinical routine. The contrast of the tumour lesions was increased after 60 min as well as 80–90 min, although a static whole-body image after 1 h still appears to be reasonable due to its ease of implementation.

To further characterise the tumour lesions in patients with BCR, this study demonstrated the feasibility of a three-compartment model and non-compartment model to calculate kinetic parameters. Bone metastases showed the strongest SUV decrease of all tumour lesions in the TAC, which was expressed in a significantly lower SUV. The kinetics of bone metastases also showed a slightly lower transport rate from the blood K_1_ and receptor binding and internalisation rate k_3_ compared to that of lymph node metastases, with a medium correlation to the SUV. The application of the three-compartment model could offer a possible explanation and characterisation of tracer uptake in the tumour lesions. However, a comparative analysis showed no statistical significance for the differences for k_3_. K_1_ could not be tested due to hierarchical testing.

The calculation of the vessel density, V_B_, was shown to be comparatively low in this work, especially in local recurrences [10, 20]. It is known from the literature that V_B_ is significantly increased in some malignant bone tumours, soft tissue tumours (sarcomas) and the semi-malignant giant cell tumours. Furthermore, a significant decrease in V_B_ is reported in immunotherapy monitoring [20]. Thus, vascular density, V_B_, could prove to be a useful parameter in diagnostics and therapy monitoring. The robustness of the calculation of this study, as well as the diagnostic value in terms of prognostic significance and therapy response, is left to further studies, partly due to the weak correlation of V_B_ with SUV.

A promising factor in this study was the FD, which was calculated with a non-compartmental model. By measuring the FD, the details of the time-activity curves were described, or more precisely, the complexity of the curves. In addition to a strong correlation with all SUVs, the FD was also significantly higher in lymph node metastases than in bone metastases. In a study by Dimitrakopoulou-Strauss et al. with FDG, it was shown in 200 tumour lesions of different entities after primary therapy that FD values > 1.13 indicate malignancy with an accuracy of 83% [22]. In metastatic melanoma, the FD could continue to be used successfully for monitoring immunotherapy, as it decreased correspondingly to therapy response [20]. Due to the high correlation with the SUV and the successful application in other tumour entities, the FD appears to be a robust and investigator-independent parameter for characterising the PSMA tracer kinetics in prostate carcinomas.

Kinetic modelling presented a simplified measurement of complex tumour biology. The potential of these functional parameters has the chance to further advance molecular imaging and improve both diagnostics and therapy management with [^68^ Ga]Ga-PSMA-11. The calculation of kinetics from dynamic measurements has also been successfully demonstrated for [^18^F]PSMA-1007 [35]. A future approach may be to use artificial intelligence to analyse the whole dataset of dynamic measurements with or without kinetic modelling. Using a large database of confirmed [^68^ Ga]Ga-PSMA-11 tumour lesions and normal tissue, an algorithm could independently find significant parameters that best characterise tumour lesions. The limitation of this research, however, could be the lower comprehensibility of the calculations, but the accuracy of the values could be even higher. In particular, a multiparametric score based on the combination of SUV, kinetic parameters K_1_ and k_3_, vessel density and fractal dimension should be evaluated in larger prospective studies for selection of patients for PSMA therapy and compared to current approaches.

### Limitations

One limitation of this study is the technically limited FOV of the dynamic acquisition of the used PET-CT scanner. Newer-generation whole-body PET-CT scanners overcome this limitation and enable whole-body dynamic acquisition including parametric imaging like Patlak analysis [36–38]. Another limitation is the lack of histopathological validation which is not possible in routine studies. Furthermore, the impact of hormone therapy was not considered due to the retrospective character of this study.

## Conclusion

[^68^ Ga]Ga-PSMA-11 PET-CT is suitable for detecting tumour lesions in biochemical recurrent prostate cancer, as the detection rate for PSA > 2 was 84%. In the difficult subgroup with 0.4 < PSA < 1, there were still 56% of patients positive on PET-CT. Dynamic imaging for visualising pharmacokinetics shows that all tumour lesions demonstrate the strongest increase in activity within the first 10 min due to a high blood clearance, which then flatten out to varying degrees. This time window can be used to acquire early images without bladder activity interfering with the differentiation of local recurrences. In this study, no further lesions were detected by early imaging; however, distinction of local recurrences was easier due to lower bladder activity. Bone metastases showed the greatest decrease in activity, as evidenced by a significantly lower SUV after 60 min compared to lymph node metastases. The application of a three-compartment model and a non-compartment model based on the calculation of the fractal dimension demonstrated a lower transport rate from the blood, lower binding and internalisation rate, significantly lower vessel density and significantly lower heterogeneity for bone metastases compared to lymph node metastases. Kinetic parameters could therefore further characterise the kinetics of tumour lesions, with heterogeneity (fractal dimension FD) in particular being a promising parameter due to its high correlation with SUV, as well as being robust and investigator-independent.

## Supplementary Information

Figure 8 Follow-up of a patient with prostate carcinoma at three different points in time. The left image shows the initial examination after electrocoagulation and a PSA of 10 ng/mL. In the whole-body MIP, in addition to physiological accumulation, some small lymph node metastases in the abdomen are visible. The middle image shows the whole-body MIP of the follow-up after chemotherapy with docetaxel six months later. At this time, the PSA was again 10 ng/mL but new tumour lesions were not detected. The known lymph node metastases showed only a faint, barely detectable uptake. The right picture shows the patient's whole-body MIP 1.5 years after the follow-up in the middle. The PSA at this time was 14 ng/mL and [68Ga]Ga-PSMA-11 PET-CT showed clear progression. Disseminated metastases are depicted in the entire body trunk, whereby in addition to known and new lymph node metastases, there is also a clear bone metastasis primarily in the thoracic spine and the right shoulder. (PNG 8794 kb)

## Data Availability

The datasets generated during and/or analysed during the current study are available from the corresponding author on reasonable request.

## Code availability

PMOD 3.7, Aycan workstation 3.14.006, Stata/MP 14.2 (StataCorp LLC).
