# Supplementary figures and images for: Pharmacokinetic studies of [68 Ga]Ga-PSMA-11 in patients with biochemical recurrence of prostate cancer: detection, differences in temporal distribution and kinetic modelling by tissue type
Source: Eur J Nucl Med Mol Imaging. 2021 Jun 10;48(13):4472–82. doi: 10.1007/s00259-021-05420-1 (PMC8566392; doi:10.1007/s00259-021-05420-1)

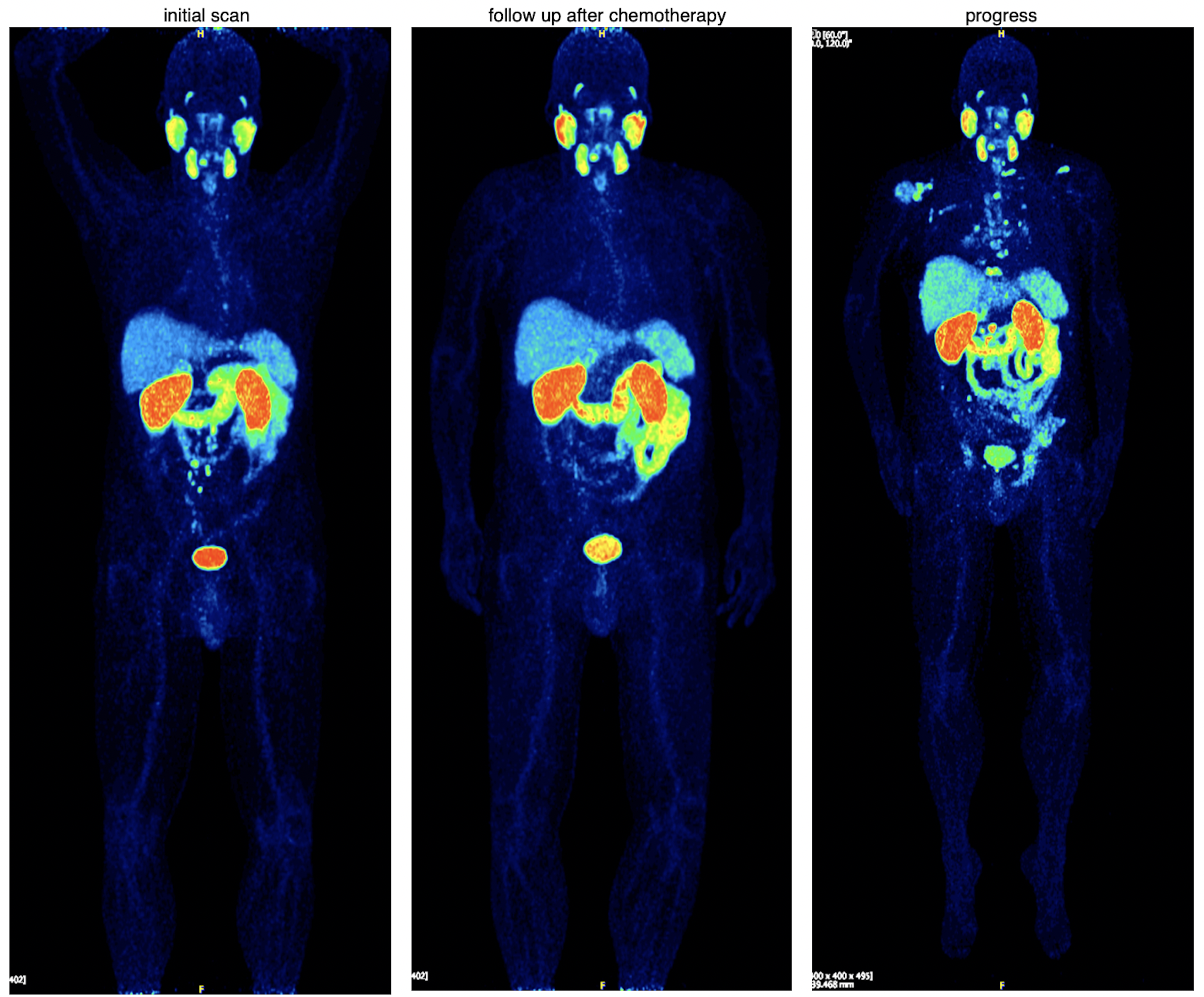

Supplement: Supplementary file 1 — Follow-up of a patient with prostate carcinoma at three different points in time. The left image shows the initial examination after electrocoagulation and a PSA of 10 ng/mL. In the whole-body MIP, in addition to physiological accumulation, some small lymph node metastases in the abdomen are visible. The middle image shows the whole-body MIP of the follow-up after chemotherapy with docetaxel six months later. At this time, the PSA was again 10 ng/mL but new tumour lesions were not detected. The known lymph node metastases showed only a faint, barely detectable uptake. The right picture shows the patient's whole-body MIP 1.5 years after the follow-up in the middle. The PSA at this time was 14 ng/mL and [68Ga]Ga-PSMA-11 PET-CT showed clear progression. Disseminated metastases are depicted in the entire body trunk, whereby in addition to known and new lymph node metastases, there is also a clear bone metastasis primarily in the thoracic spine and the right shoulder. (PNG 8794 kb) [file 259_2021_5420_MOESM1_ESM.png]
